# Theoretical study of the role of metallic contacts in probing transport features of pure and defected graphene nanoribbons

**DOI:** 10.1186/1556-276X-6-234

**Published:** 2011-03-18

**Authors:** Antonino La Magna, Ioannis Deretzis

**Affiliations:** 1 1CNR IMM, Z.I. VIII Strada 5, 95121 Catania, Italy

## Abstract

Understanding the roles of disorder and metal/graphene interface on the electronic and transport properties of graphene-based systems is crucial for a consistent analysis of the data deriving from experimental measurements. The present work is devoted to the detailed study of graphene nanoribbon systems by means of self-consistent quantum transport calculations. The computational formalism is based on a coupled Schrödinger/Poisson approach that respects both chemistry and electrostatics, applied to pure/defected graphene nanoribbons (ideally or end-contacted by various fcc metals). We theoretically characterize the formation of metal-graphene junctions as well as the effects of backscattering due to the presence of vacancies and impurities. Our results evidence that disorder can infer significant alterations on the conduction process, giving rise to mobility gaps in the conductance distribution. Moreover, we show the importance of metal-graphene coupling that gives rise to doping-related phenomena and a degradation of conductance quantization characteristics.

## Introduction

Graphene nanoribbons (GNRs) are the most promising graphene-based nanostructures for electronic applications since they are potentially suited for band-gap engineering, maintaining the excellent electronic properties of the parent two-dimensional graphene layer. GNRs have been already synthesized by means of different pattering techniques [1,2], and there exists convincing evidence that their electronic structure manifests subband formation which is a typical predicted signature of the one-dimensional (1D) confinement [3]. Actually, a useful intrinsic (i.e., due to the geometric confinement) band-gap value can be obtained only for GNR structures with widths of approximately 1 nm that cannot be easily fabricated with the current lithographic techniques. As a consequence, the controlled inclusion of defects and/or impurities in GNRs has been proposed to overcome the minimum of conductivity problem [4]. Anyhow, we should assume that unintentionally defected GNRs are present from the phase of production due to the impossibility of an accurate control of purity at an atomic level.

Electrical characterization studies of GNRs must preliminarily consider the impact of disorder in their electrical properties, which is crucial also due the possible Anderson localization phenomena in these quasi-1D systems [5]. Moreover, the formation of a junction between a relatively large metal probes (i.e., a three-dimensional (3D) system) and GNRs (a 1D system) is at the basis of any electrical measurements. Understanding the role of interface bonding and electrostatics in the contact region is also crucial in order to categorize the electronic characteristics of these systems. Indeed, we could argue that in the absence of defects, a significant source of resistivity in GNRs and the consequent deviations from the ideal behavior should be derived from the interaction with the metallic electrodes. In this work, these issues are theoretically investigated and their implications on the interpretation of electrical measurements on GNR-based systems are discussed.

## Theoretical approach

We consider pure and defected hydrogen-terminated armchair GNRs (AGNR) of different widths and lengths (from few nanometers to approximately a micrometer) with two different contact configurations: (a) ideally contacted (same width as the conductor without defect or impurity inclusions) at the right side and the left side, and (b) end-contacted by 3D semi-infinite metallic electrodes (Au, Pd, and Al (111) surfaces) at the left side whereas ideally contacted at the right side. The terminology of Ref. [6] is applied to categorize them on the basis of the dimer lines *N*_a _along the ribbon width (e.g., with *N*_a _= 45 AGNR, we indicate a ribbon with 45 dimer lines). We use the non-equilibrium Green function (NEGF)(1)

and the standard Landauer-Buttiker approach for the calculation of quantum transport [7]. Hamiltonians *H*_0 _and overlap matrices *τ *are written within first-principles parameterized models [8].

Metal surface Green functions *g*_s _for the evaluation of the respective self-energies Σ = τ *g*_s_τ^† ^are calculated for the 3D semi-infinite contact with a back and forth real to *k*-space Fourier transform exploiting lattice periodicity [8]. Mobile charges *ρ*_f _deriving from the NEGF are passed to a 3D numerical Poisson solver from where the self-consistent potentials are calculated by solving ∇^2^*U*_SC _= -*ρ*_f_*/*ε, where *H = H*_0 _*+ U*_SC _in Equation 1. Electrical potential has been fixed at the metal-GNR interface (applying a Dirichelet type boundary condition) at a value *U*_left __SC _*= φ*_m _- *φ*_gr_, where *φ*_m _and *φ*_gr _are the experimentally measured work functions for (111) metallic surfaces and graphene [8], while zero electric field (i.e., null Neumann boundary condition) has been set for the ideal contacts. Self-consistency is enhanced by a predictor/corrector Newton-Rapson algorithm. Fermi-Dirac statistics have been introduced in the simulation scheme for temperature *T *= 300 K.

## Defected GNRs

Isolated defect and impurity alter both the density of states distribution DOS(*E*) than conductance *g*(*E*) of quasi-1D GNRs. In particular n-type (p-type) impurities introduce electronic states (also called resonance states [3]) at energies above (below) the charge neutrality point (i.e., the Dirac point) of the pristine pure systems. In correspondence of these states, a decrease of the conductance with respect the pure system can occur because of the scattering of the conducting electron by the effective potential due to the impurity presence [3]. The conductance alteration strongly depends on the position of the scattering center in the nanoribbon structure [8]. However, in general, we expect that defective GNRs are characterized by larger density of the states and smaller conductance with respect to the ideally pure GNRs.

**Figure 1:**
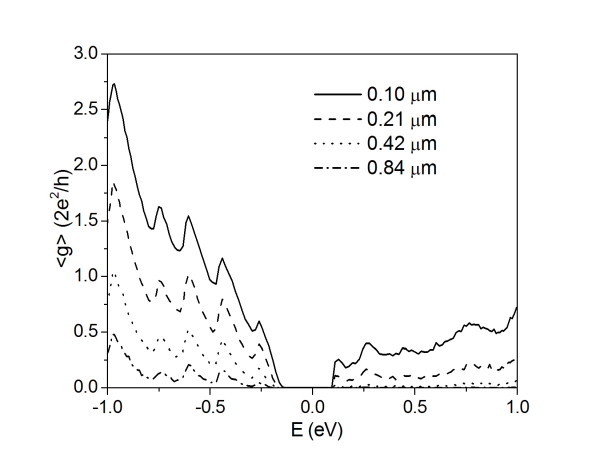
**Average conductance for a nitrogen-doped *N*_a _= 45 AGNR**. Average conductance <g> as a function of the energy *E *for a nitrogen-doped *N*_a _= 45 AGNR of different lengths. Plotted values represent statistical averages over more than 500 equivalent replicas of the system. Charge neutrality points of pure and doped systems are aligned at *E *= 0 eV in the figure.

In a real defected system, we expect a finite density of random distributed scattering centers and, as a consequence, the effect of multiple scattering processes should be evaluated in this kind of configuration. In Figure 1, we show the (small bias) average conductance of an ideally contacted *N*_a _= 45 AGNR doped with a 0.2% density of nitrogen atoms and for systems with increasing length *L*, from approximately 0.1 μm to approximately 0.8 μm. An asymmetric decrease of the average conductance due to the impurity scattering can be observed for the whole spectrum. This behavior is particularly important in the energy region near the single impurity resonance states (i.e., for energies *E *≈ 0.2 eV) where a mobility gap appears also for the smaller systems. We note that the pure *N*_a _= 45 AGNR is a semiconductor GNR with an energy gap of approximately 0.2 eV.

**Figure 2:**
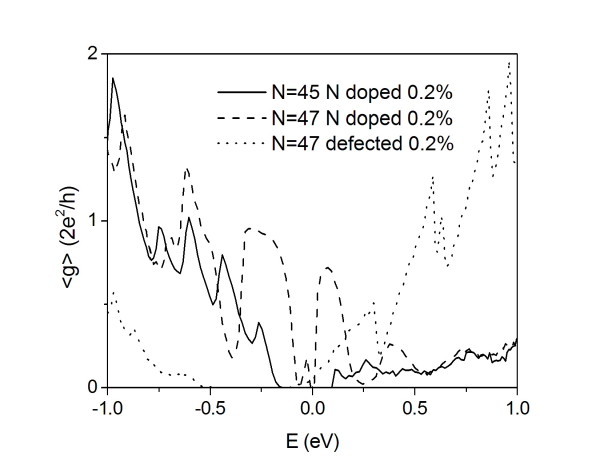
**Average conductance for nitrogen-doped and vacancy-damaged AGNRs**. Average conductance <g> as a function of the energy *E *for a nitrogen-doped *N*_a _= 45 AGNR (solid line), nitrogen-doped *N*_a _= 47 AGNR (dashed line), and vacancy-damaged *N*_a _= 47 AGNR (point) with fixed length: *L *≈ 0.21 μm. Plotted values represent statistical averages over more of 500 equivalent replicas of the system. Charge neutrality points of pure and defected systems are aligned at *E *= 0 eV in the figure.

A qualitatively similar behavior is shown by the vacancy-damaged and nitrogen-doped semimetallic *N*_a _= 47 AGNR (see Figure 2). In the vacancy-damaged case, a large mobility gap appears in the negative energies (hole band) region [9] due to the strong backscattering of the defects. However, apart from the intensity of the scattering, vacancy-defected systems have a p impurity-like behavior.

**Figure 3:**
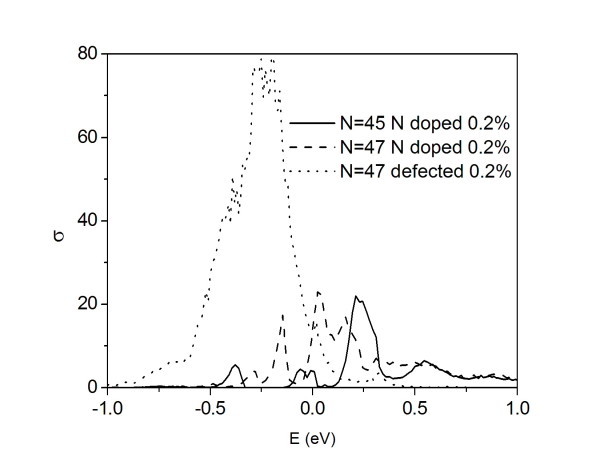
**Conductance fluctuation for nitrogen-doped and vacancy-damaged AGNRs**. Conductance fluctuation *σ *as a function of the energy *E *for a nitrogen-doped *N*_a _= 45 AGNR (solid line), a nitrogen-doped *N*_a _= 47 AGNR (dashed line), and a vacancy-damaged *N*_a _= 47 AGNR (point) with fixed length: *L*~0.21 μm. Plotted values represent statistical averages over more of 500 equivalent replicas of the system. Charge neutrality points of pure and defected systems are aligned at *E *= 0 in the figure.

From an analysis of the conductance spectra, we can derive same general features which can be useful for the interpretation of the electrical characterization of real GNRs. Indeed, we note that the conduction spectrum can be measured in a three terminal configuration [4] tuning the gate potential in order to modify the electron density in the nanostructure. Firstly, we note the persistence of the conduction modulation with energy in disordered systems, which is a marker of the conductance plateaus of the subband structure in pure GNRs. The effective transmission in the subbands is strongly reduced due to localization effects that suppress the conductance transparency in the spectral region where resonance states are located. Finally, the general occurrence of mobility gaps (see also Ref. [10]) can be hardly distinguished from the intrinsic bandgaps in semiconductor GNRs when experimentally measuring the conductance. A clear signature of the mobility gap is the occurrence of huge values of conductance fluctuations in the same spectral region of the gap due to its backscattering origin. In order to demonstrate this assumption, we have plotted in Figure 3 the statistically evaluated conductance variance

(where *g *is in 2*e*^2^/*h *units) for the same cases of Figure 2. In the quasi-metal systems, the conduction depletions are systematically related to variance peaks, while the variance in the standard gap of the semiconductor GNRs does not show particular features.

## Metal-GNR junction

Self-consistent local density of the state (LDOS) calculations have shown a non-conventional scenario for the charging and the related electrostatics of the heterojunction between the 3D metal and the 1D GNRs [8]. In particular:

(a) The electrostatic potential, due to the work function difference, drops in a few-nanometer region near the interface and has non-zero flat value, denoting the presence of carrier accumulation throughout the GNR length;

(b) Unlike the standard metal semiconductor interface, band bending is not rigid for both conduction and valence bands as a result of quantum interference (between wave functions with 1D character in the nanoribbon and 3D character in the metal) and electron confinement. For example, in the case of a metal with high (low) work function with respect to the graphene, like Au (Al), conduction (valence) band states shift smoothly following the electrostatic potential, while discrete localized states appear with a few-nanometer spatial extension in the region where the valence (conduction) band maximally bends;

(c) Quantum interference, localized states, and the metal induced gap states (i.e., the tails of the metal wavefunctions) characterize the LDOS in the junction region.

**Figure 4:**
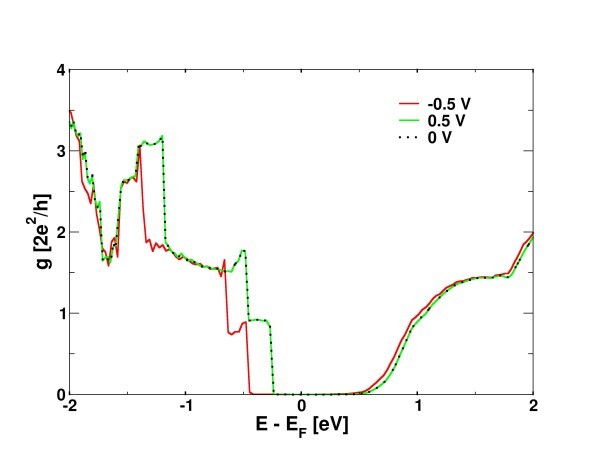
**Small- and finite-bias conductance spectra**. Small-bias (points) and finite-bias (+0.5 V green line, -0.5 red line) conductances of a pure *N*_a _= 16 AGNR end-contacted with Au.

The local electronic structure characteristics in the contact region can non-trivially influence the conduction mechanism since, e.g., localized states do not contribute to the conduction, giving rise to conductance asymmetries and an overall loss of the transport information with respect to the ideal case. In Figure 4, small- and finite-bias conductance spectra of a pure *N_a _= *16 AGNR end-contacted with Au are plotted as a function of energy. These spectra show similar characteristics with the ones obtained considering metals with higher work functions than graphene (e.g., Pt, Pd). A Schottky barrier of the order of 0.2 to 0.3 eV can be determined by the difference between the gap in the conductance spectrum for the contacted GNRs at 0 V (Figure 4 dotted line) and the gap (approximately 0.6 eV) of the ideal non-contacted GNR.

Apart from the Schottky barriers, we note that conduction band charge flow is strongly suppressed due to the band bending and the p-type character, giving an asymmetric form to the overall conductance distribution (asymmetric spectra, not shown here, are calculated also for quasi-metallic GNRs ). Note that finite-bias conductance spectra are affected by the presence of the Schottky barrier. Indeed, while the -0.5-V case shows the alteration with respect to the small-bias case due to non-equilibrium charging (see Ref. [11] for a complete discussion), the +0.5 is almost identical to the small-bias case.

**Figure 5:**
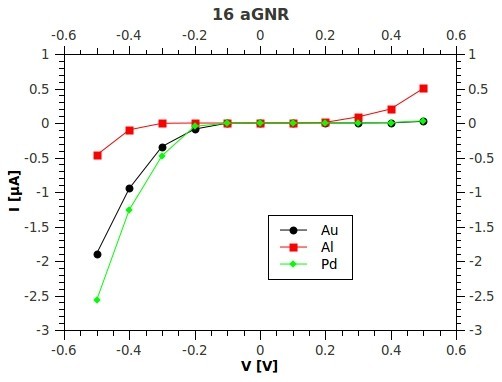
**Current-voltage characteristics**. *I*-*V *characteristics derived by the NEGF in the Landauer-Buttiker scheme for a *N*_a _= 16 AGNR, end-contacted with three different metals Au (black line), Pd (green line), and Al (red line).

Current-voltage (*I*-*V*) characteristics of the junction in the case of a pure *N_a _= *16 AGNR contacted with different metals are reported in Figure 5. Larger *I *values obtained for a negative bias in the case of Pd with respect Au are due to its slighter more pronounced p-type character (while in turn Au seems transparent near the Fermi level with the conductance arriving at the 1 *G*_0 _*= *2*e*^2^/*h *plateau of the ideal case). Al has a lower work function with respect to graphene and the Al-GNR junction shows a quasi-ambipolar Schottky behavior (i.e., the *I*-*V *characteristic is almost symmetric for positive and negative bias). However, in the latter case, the dominant aspect is the strong scattering by the contacts and the related suppression of the contact transparency.

## Conclusion

The results of this theoretical study have shown that defects, doping, and metal/graphene interfaces can non-trivially influence the conduction mechanism of graphene nanostructures, giving rise to mode transmission suppression, conductance asymmetries, mobility gap formations, and strong conductance fluctuations. The implication of these results for the electrical characterization of real structures has been discussed. A general crucial issue, which should also be taken into account when probing transport in GNRs, is that the electrostatics and the chemical bonding aspects can act complementary for the determination of transparency in graphene-based nanostructures.

## Competing interests

The authors declare that they have no competing interests.

## Authors' contributions section

AL carried out the conduction calculation and the statistical analysis for the defective system. ID carried out all the *ab-inito *based calculation presented in this manuscript. AL and ID developed the formalism used for conduction evaluation.
